# 3,3′-Diindolylmethane inhibits the invasion and metastasis of nasopharyngeal carcinoma cells *in vitro* and *in vivo* by regulation of epithelial mesenchymal transition

**DOI:** 10.3892/etm.2014.1649

**Published:** 2014-03-28

**Authors:** TINGTING WU, CHEN CHEN, FEN LI, ZHE CHEN, YONG XU, BOTUI XIAO, ZEZHANG TAO

**Affiliations:** Department of Otolaryngology-Head and Neck Surgery, Renmin Hospital of Wuhan University, Wuhan, Hubei 430060, P.R. China

**Keywords:** 3,3′-diindolylmethane, nasopharyngeal carcinoma cell, invasion, metastasis, epithelial mesenchymal transition

## Abstract

Nasopharyngeal carcinoma (NPC) is characterized by silent progression and atypical early symptoms. Early metastasis to the neck lymph nodes is common. However, conventional chemoradiotherapy is limited and unable to effectively control cervical lymph node metastasis of NPC. In addition, toxicities caused by chemoradiotherapy often induce damage to normal tissues and organs. Thus, the aim of this study was to investigate the ability of 3,3′-diindolylmethane (DIM) to inhibit the invasion and metastasis of NPC cells *in vitro* and *in vivo*. The migration and invasive abilities of the 5–8F human NPC cell line were detected using a Transwell assay. Lymph node metastasis in nude mice was observed following the implantation of xenograft tumors for 8 weeks. In addition, western blot analysis was used to detect the expression levels of epithelial mesenchymal transition (EMT)-associated key proteins in NPC cells treated with DIM *in vitro* and *in vivo*. The results demonstrated that DIM effectively inhibited the migration and invasion of NPC cells *in vitro* and the effect was concentration-dependent. In addition, DIM significantly delayed and reduced the occurrence of lymph node metastasis in the animal model. The expression levels of a number of key proteins associated with EMT were affected by DIM treatment. In the animal model, there were no signs of toxicity in the vital organs, including the heart, liver and kidney, of animals fed a diet containing DIM. Therefore, the results of the present study indicate that DIM affects the expression levels of a number of EMT-associated key proteins and induces the inhibition of invasion and metastasis of NPC cells *in vitro* and *in vivo*.

## Introduction

Nasopharyngeal carcinoma (NPC) is a common malignancy with a high metastatic and invasive rate that originates in the epithelial lining of the nasopharynx. NPC is most prevalent in southeast Asia, parts of Africa and southern China (1,2) and there were an estimated 84,400 cases of NPC and 51,600 mortalities in 2008 worldwide (3). Since NPC is radiosensitive, radiotherapy serves as the primary treatment, achieving 5-year overall survival rates of 90 and 84% for early stage I and IIA NPC, respectively (4). Although NPC is sensitive to radiotherapy and chemotherapy, the majority of patients are diagnosed during the middle-late stage and despite local lesions being well controlled, treatments for lymph node metastasis and local recurrence are limited. A number of clinical studies (5,6) have found that the nodal status is an independent prognostic factor that affects the overall survival rate of NPC patients without distant metastasis. However, for advanced NPC, treatment outcomes have been unsatisfactory due to distant metastases and local recurrence following radiotherapy (7). The clinical use of chemoradiotherapy is often limited by unacceptable levels of normal tissue toxicity (8). Thus, more effective treatment methods and drugs are required to effectively control lymph node metastasis and improve the overall survival rate (9).

Crucifers, including broccoli, cauliflower and carrots, are plants with natural antitumor activity. 3,3′-Diindolylmethane (DIM) is a specific compound extracted from crucifers that has been identified to provide the main antitumor function. A number of studies (10–13) have reported that DIM inhibits proliferation and induces apoptosis in a variety of tumor cells *in vitro* and *in vivo*. In a previous study (14), DIM was found to effectively inhibit the proliferation of NPC cells and induce them to undergo apoptosis. Multiple signaling pathways, including the phosphoinositide-3-kinase, protein kinase B, mitogen activated protein kinase and nuclear factor-κB pathways, were shown to be regulated by DIM and the expression of key proteins associated with these pathways was also shown to be inhibited. In addition, no signs of toxicity were observed in the normal tissues and organs in the animal model, indicating that DIM is safe to use as an antitumor drug. The study concluded that DIM effectively induced apoptosis of NPC cells and had a preventive and curative role in the development and progression of NPC.

However, whether DIM inhibits lymph node metastasis of NPC cells remains to be investigated. Thus, the aim of the present study was to explore whether DIM has the ability to inhibit the lymph node metastasis of NPC cells. The molecular mechanism was also investigated.

## Materials and methods

### Cell culture and grouping

The 5–8F Human NPC cell line (China Center for Type Culture Collection, Wuhan University, Wuhan, China) was cultured in RMPI-1640 (Invitrogen Life Technologies, Carlsbad, CA, USA) with 10% fetal bovine serum (FBS; HyClone Laboratories, Inc., South Logan, UT, USA) in an environment of 5% CO_2_ and 37°C. Cells in a logarithmic growth phase were used in the experiment. DIM, at final concentrations of 0, 25, 50 and 100 μM, was added to the 5–8F cell cultures.

### Transwell assay

Transwell assays were performed using polycarbonate membrane Transwell plates coated with Matrigel (Corning Inc., Tewksbury, MA, USA). The bottom chamber included 0.5 ml medium containing 5% FBS. Cells were seeded into the upper chamber at a density of 3.0×10^5^ cells/ml and incubated for 24 h at 37°C and 5% CO_2_. The remaining cells on the upper surface were mechanically removed. Membranes were then washed, fixed and stained with methyl violet. Using a microscope, the migration ability of the cells was determined by counting the cells that had migrated to the lower side of the filter. Experiments were performed in triplicate and three fields were counted in each experiment.

### Animal feeding and grouping

Female BALB/c nude mice (age, 4–6 weeks-old) were purchased from Beijing Huafukang Biological Technology Co. Ltd. (Beijing, China). The animals were fed the control diet for one week prior to the experiment in order to adapt. A conventional diet and 0.5% DIM-supplemented diet were manufactured by Beijing Huafukang Biological Technology Co. Ltd. Animal welfare and transplanted tumor inoculation procedures were performed as previously described (14). Animals were divided into three groups. The preventive treatment group received a 0.5% DIM-supplemented diet prior to the inoculation of NPC cells. The drug treatment group received the 0.5% DIM-supplemented diet following the inoculation of NPC cells and the tumor diameter reaching 5 mm, while the control group was inoculated with NPC cells and fed a control diet. The duration of DIM treatment was 8 weeks and the time of lymph node metastasis occurrence was observed and recorded. Eight weeks after inoculation, the animals were sacrificed and the xenografts, metastatic lymph nodes, hearts, livers and kidneys were preserved for further testing. The study was conducted in strict accordance with the recommendations in the Guide for the Care and Use of Laboratory Animals of the National Institutes of Health (eighth edition). The animal use protocol was reviewed and approved by the Institutional Animal Care and Use Committee of Wuhan University (Wuhan, China).

### Western blot analysis

Cells were stimulated with various concentrations of DIM, harvested and then lysed in buffer containing 1% Nonidet-P40 supplemented with complete protease inhibitor cocktail (Roche Diagnostics, Mannheim, Germany) and 2 mM dithiothreitol. Lysates were resolved by 12% SDS-PAGE, transferred to nitrocellulose membranes and immunoblotted with primary antibodies against E-cadherin, matrix metalloproteinase (MMP)-9, vimentin, slug, snail and GAPDH (Cell Signaling Technology, Inc., Danvers, MA, USA). Following immunoblotting with secondary antibodies (Beijing Zhongshan Golden Bridge Biotechnology Co., Ltd., Beijing, China), proteins were detected with enhanced chemiluminescence reagent (Thermo Fisher Scientific, Waltham, MA, USA). All experiments were performed in triplicate.

### Statistical analysis

All values are expressed as mean ± SD. Statistical analysis was conducted using one-way analysis of variance with SPSS statistical software version 15.0 (SPSS, Inc., Chicago, IL, USA). P<0.05 was considered to indicate a statistically significant difference.

## Results

### Invasive ability of NPC cells in vitro

**Figure 1:**
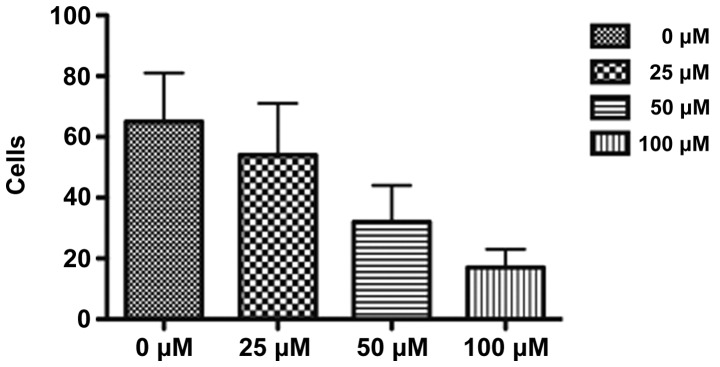
DIM inhibits the invasion ability of 5–8F NPC cells. Transwell Matrigel invasion assays were performed and the average number of invading cells, determined in three independent experiments, are shown in each column. The number of cells migrating across the membrane decreased at the concentration of DIM increased. NPC, nasopharyngeal carcinoma; DIM, 3,3′-diindolylmethane.

The migration and invasive abilities of NPC cells were detected using the Transwell assay. The results demonstrated that NPC cells treated with DIM had a decreased ability to migrate and invade. In addition, the weakening effect was increased as the concentration of DIM increased (Fig. 1).

### Invasive ability of NPC cells in vivo

In the control group, lymph node metastasis occurred 4 weeks following the implantation of NPC cells, and lymph node metastasis was observed in 10/12 rats at week 8 following implantation. In the drug treatment group, lymph node metastasis occurred 6 weeks following implantation and lymph node metastasis was observed in 6/12 rats at week 8 following implantation. In the preventive treatment group, lymph node metastasis occurred 7 weeks following implantation and lymph node metastasis was observed in 2/12 rats at week 8 following implantation. The differences between the three groups were statistically significant (Table I).

### Expression of key proteins associated with epithelial mesenchymal transition (EMT)

**Figure 2:**
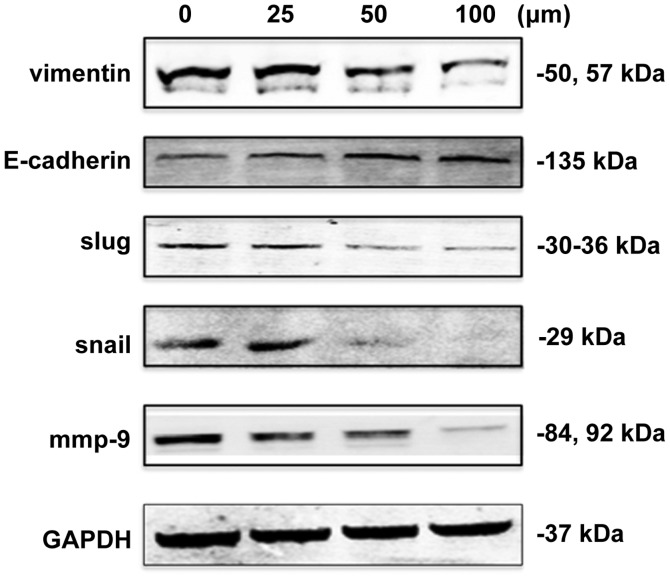
Expression of EMT-associated proteins in 5–8F NPC cells treated with various concentrations of DIM. Expression levels of the EMT-associated proteins vimentin, E-cadherin, slug, snail and MMP-9 were detected by western blot analysis. The expression levels of vimentin, slug, snail and MMP-9 decreased with increasing concentrations of DIM, while the expression levels of E-cadherin increased with increasing concentrations of DIM. NPC, nasopharyngeal carcinoma; DIM, 3,3′-diindolylmethane; EMT, epithelial mesenchymal transition.

Expression levels of EMT-associated key proteins in NPC cells treated with DIM were detected to investigate the molecular mechanisms underlying the inhibition of invasion and metastasis *in vitro* and *in vivo*. The results demonstrated that the protein expression level of E-cadherin increased gradually with increasing concentrations of DIM. By contrast, vimentin, slug and snail expression levels gradually decreased (Fig. 2).

## Discussion

The majority of patients with NPC are found to have a palpable mass in their neck following physical examination. Once neck lymph node metastasis has occurred, the treatment outcomes of NPC are poor (15). This is mainly due to the limitations of conventional chemoradiotherapy treatment in controlling lymph node metastasis (16). In previous years, although radiation technology has developed and a variety of new chemotherapy drugs have been applied in clinical practice, the 5-year overall survival rate of NPC patients has not significantly improved (17–23).

A number of studies (24–27) have demonstrated that EMT plays a key role in tumor development. The migration capacity of tumor cells is associated with cell phenotype. EMT is typically characterized by spindle-shaped cells that are unable to form homophilic cell-cell interactions due to the addition of a vimentin cytoskeleton and the loss of E-cadherin (28,29). Tumor cells with a mesenchymal phenotype, in contrast to epithelial tumor cells, have higher expression levels of cell movement-associated proteins and exhibit improved migration and invasion (30). The results of the present study indicate that lymph node metastasis of NPC was significantly inhibited by DIM. To the best of our knowledge, the function of DIM in inhibiting lymph node metastasis has not been previously reported. The molecular mechanism of DIM was considered to involve inhibition of the EMT phenotype of NPC cells.

E-cadherin and vimentin are important markers in EMT (31–33). E-cadherin is an epithelial cell marker, while vimentin is a mesenchymal cells marker. Wong *et al* (34) reported that E-cadherin expression levels decreased in NPC cells undergoing migration. In addition, the migration potential of NPC cells significantly decreased when exogenous E-cadherin was expressed, according to gene technology. Xu *et al* (35) found that E-cadherin expression exhibited a significant positive correlation with the long-term outcome of NPC patients; that is, the higher the E-cadherin expression levels, the better the long-term outcome of patients. Among the NPC patients who received the antitumor therapy, the reoccurrence and lymph node metastasis rates of the patients with higher E-cadherin expression were lower than those with lower E-cadherin expression. The study concluded that EMT is one of the factors that affects the long-term outcomes of patients with NPC. In the present study, the NPC cell line 5–8F had a marked capability for invasion and were identified to have an EMT phenotype with low E-cadherin expression levels and high vimentin expression levels. However, the invasion ability of 5–8F cells decreased markedly following treatment with DIM and the E-cadherin expression levels increased with increasing concentrations of DIM. In addition, the expression levels of vimentin decreased with increasing concentrations of DIM. In the animal model, lymph node metastasis occurrence was reduced and delayed in rats fed the 0.5% DIM-supplemented diet compared with that in the control group. These results indicate that DIM significantly inhibits the invasion ability and lymph node metastasis of NPC cells.

Slug and snail are transcription factors that play an important role in tumor development as they inhibit E-cadherin expression and promote EMT (36,37). It has been reported that by inhibiting slug and snail, which in turn inhibit EMT, the invasion and metastasis of tumor cells is inhibited, causing the expression levels of E-cadherin to increase. In the present study, the expression levels of slug and snail in the NPC cells treated with DIM were detected and found to decrease with increasing concentrations of DIM. Therefore, it may be hypothesized that the reductions in the slug and snail expression levels caused by DIM were associated with the inhibition of invasion and metastasis of the NPC cells, and that the inhibition of invasion and metastasis was directly caused by reductions in the E-cadherin and vimentin expression levels.

The control of lymph node metastasis is crucial in the treatment of NPC. The expression levels of key markers of the EMT phenotype have been shown to directly correlate with the prognosis of patients with NPC (38). In the present study, a natural and non-toxic compound extracted from plants was shown to effectively inhibit the invasion and metastasis of NPC cells by inhibiting EMT *in vivo* and *in vitro*. However, the treatment effects of DIM combined with conventional chemotherapy and radiotherapy, as well as the function of inhibiting EMT by DIM for use in clinical practice, require further study.

In conclusion, DIM affects the expression levels of a number of EMT-associated key proteins and effectively induces the inhibition of invasion and metastasis of NPC cells *in vitro* and *in vivo*.

## Figures and Tables

**Table I:** Incidence of xenograft tumor and lymph node metastasis (n=12 per group).

Groups	Xenograft tumor, n (%)	Lymph node metastasis, n (%)
Control	12/12 (100)	10/12 (83.3)
Preventive treatment	9/12 (75)	2/9 (22.2)
Drug treatment	10/12 (83.3)	6/10 (60)
